# Rapid Detection of Plasticizer Migration From UV‐Aged PVC Films by DART‐HRMS

**DOI:** 10.1002/rcm.70048

**Published:** 2026-02-17

**Authors:** Odilon Leite‐Barbosa, Marcelo Ferreira Leão de Oliveira, Márcia Gomes de Oliveira, Monica Costa Padilha, Valdir Florêncio Veiga‐Junior

**Affiliations:** ^1^ Materials Engineering Department Military Institute of Engineering Rio de Janeiro Rio de Janeiro Brazil; ^2^ National Institute of Technology (INT), division of Materials Rio de Janeiro Rio de Janeiro Brazil; ^3^ Brazilian Doping Control Laboratory (LBCD), Chemistry Institute Federal University of Rio de Janeiro (UFRJ) Rio de Janeiro Rio de Janeiro Brazil

**Keywords:** ambient ionization, DART‐HRMS, phthalates, plasticizer migration, poly(vinyl chloride), PVC aging

## Abstract

**Rationale:**

The migration of phthalate plasticizers from high‐volume polymers, such as poly(vinyl chloride) (PVC), raises significant toxicological concerns, particularly when materials are subjected to aging and environmental stress. As traditional monitoring techniques rely on time‐consuming solvent extraction and chromatographic methods that are often expensive and lack the throughput required for large‐scale safety screening, this study intends to present a rapid screening method using direct analysis in real time coupled to high‐resolution mass spectrometry (DART‐HRMS) to monitor plasticizer migration in PVC films subjected to accelerated UV aging. Operational parameters, such as ionization gas temperature and grid voltage, were systematically optimized to balance desorption efficiency with molecular integrity.

**Methods:**

DART‐HRMS analysis of three commercial PVC films subjected to accelerated UV aging (ASTM G154‐23) was performed after optimization of the operational parameters: ionization gas temperature: 250 °C, 350 °C, and 500 °C and grid voltage: 50 and 350 V were systematically optimized to balance desorption efficiency with molecular integrity.

**Results:**

Analytical performance was strongly governed by source energy, with optimal conditions achieved at 350 °C and 50 V, yielding the highest signal stability and minimal in‐source fragmentation. Elevated grid voltage (350 V) caused severe signal suppression and fragmentation, particularly for high‐molecular‐weight plasticizers such as DIDP and DINP. Application of the optimized method revealed formulation‐dependent migration behavior during UV aging. Short‐chain phthalates showed rapid and, in some cases, transient surface enrichment, whereas medium‐ and high‐molecular‐weight plasticizers exhibited delayed or limited migration, becoming detectable only after prolonged exposure.

**Conclusions:**

DART‐HRMS provides a fast, robust, and solvent‐free approach for screening plasticizer migration in PVC films. The optimized conditions enable sensitive detection while preserving molecular integrity, allowing differentiation of additive mobility as a function of molecular weight, formulation, and UV‐induced degradation. This methodology offers a high‐throughput alternative for assessing additive stability and potential release in consumer‐grade PVC materials.

## 1 Introduction

PVC remains one of the most extensively used thermoplastics worldwide, and its versatility is especially exploited in flexible consumer products such as films, tubing, and soft packaging. In such applications, PVC is heavily formulated with external plasticizers, primarily phthalate esters, which impart critical properties like flexibility, transparency, and long‐term mechanical stability to otherwise rigid polymer chains [1, 2]. Because plasticizers typically constitute a substantial fraction of the final material mass, their chemical behavior and potential migration under environmental or use conditions play a key role in determining the functional lifetime and safety profile of PVC‐based products [3, 4].

A key challenge inherent to plasticized poly(vinyl chloride) (PVC) lies in the absence of covalent bonds between the polymer backbone and the phthalate‐based external plasticizers, which renders the material especially vulnerable to mass‐transport phenomena under environmental stressors such as heat, oxygen, or ultraviolet (UV) radiation [5, 6]. Under UV exposure, plasticized PVC undergoes photodegradation processes, notably dehydrochlorination, chain scission, and oxidative reactions that disrupt the polymer microstructure and increase free volume, thereby greatly facilitating the upward diffusion and leaching of plasticizers toward the surface [7, 8]. Consequently, surface‐level transformations, such as changes in surface chemistry, increased hydrophilicity, yellowing, or embrittlement, may signal early stages of material degradation, loss of mechanical performance, or even the release of plasticizer‐derived compounds with toxicological relevance [3, 8].

The migration of plasticizers from PVC raises serious toxicological and regulatory concerns. According to Systemiq, these “invisible ingredients” are a major driver of noncommunicable diseases and fertility loss, imposing a global health burden estimated between $1.4 and $2.2 trillion annually. This scenario has led to a proactive search by industries for high‐throughput screening methods to ensure both regulatory compliance and consumer safety, particularly to avoid “regrettable substitutions,” where one hazardous compound is replaced by another with similar toxic effects [9]. Certain phthalate esters frequently used as plasticizers, for example, dibutyl phthalate (DBP), bis(2‐ethylhexyl) phthalate (DEHP), diisononyl phthalate (DINP), and diisodecyl phthalate (DIDP), have been linked to endocrine disruption, reproductive toxicity, carcinogenic potential, and developmental effects such as metabolic disorders in animal models and epidemiological observations [10, 11]. Such risks are particularly relevant when plasticized PVC is used in food‐contact materials, medical devices, or consumer articles accessible to vulnerable populations such as children, as leaching or migration may lead to human exposure over time through dietary and nondietary ingestion [11, 12]. In response, regulatory bodies worldwide, including the European Food Safety Authority (EFSA) and US Consumer Product Safety Commission (CPSC), have imposed strict restrictions or bans on the use of several of these phthalates in toys, childcare articles, and materials intended for food contact [13, 14]. Such regulatory pressure underscores the urgent need for rapid, reliable analytical screening methods capable of detecting surface‐available plasticizers or assessing plasticizer mobility, especially considering the emerging evidence of adverse effects even at low‐dose exposures, a key step to ensure both compliance and safety [15].

Monitoring surface‐accessible additives in PVC has traditionally relied on solvent extraction followed by chromatographic separation and mass spectrometry, a robust but time‐consuming strategy with low spatial resolution, limiting its ability to detect local chemical changes at the surface level that may result from aging, weathering, or environmental stress [16]. Ambient ionization mass spectrometry, particularly DART‐HRMS, has recently emerged as a powerful alternative, enabling the direct desorption and ionization of analytes from solid polymer surfaces under ambient conditions with little or no sample preparation. This approach drastically reduces analysis time and preserves sample integrity [17, 18]. DART‐HRMS is particularly advantageous for polymers containing low‐volatility plasticizers or surface‐enriched additive species, as the ionization process can probe molecules that are difficult to extract or detect by conventional solvent extraction methods [16].

Significant methodological progress has been achieved in the application of DART‐HRMS for the analysis of PVC matrices. Previous investigations have successfully validated the technique as a robust tool for rapid, nondestructive screening of restricted phthalates in consumer goods such as toys and food‐contact gaskets, demonstrating its ability to detect complex mixtures of additives within regulatory limits [19, 20]. Furthermore, identification strategies have been refined by optimizing collision energies based on molecular weight, enabling automated data acquisition for phthalate esters [21]. Despite these advances in static characterization and instrumental tuning, no study to date has systematically investigated how aging processes influence plasticizer migration and ionic signal behavior in PVC materials using DART‐HRMS. However, it is known that UV irradiation, thermal cycling, and humidity variations trigger dehydrochlorination, surface oxidation, and phase segregation in PVC, which can alter the distribution and volatility of incorporated plasticizers, thus impacting their desorption and detectability in ambient ionization systems [22, 23]. The lack of validated experimental structures that integrate controlled aging with DART‐HRMS signal tracking limits our understanding of how additive intensity patterns evolve in situ, a critical gap that restricts the technique's applicability for studying migration kinetics, degradation pathways, and potential release hazards in consumer‐grade PVC films [24, 25].

**FIGURE 1:**
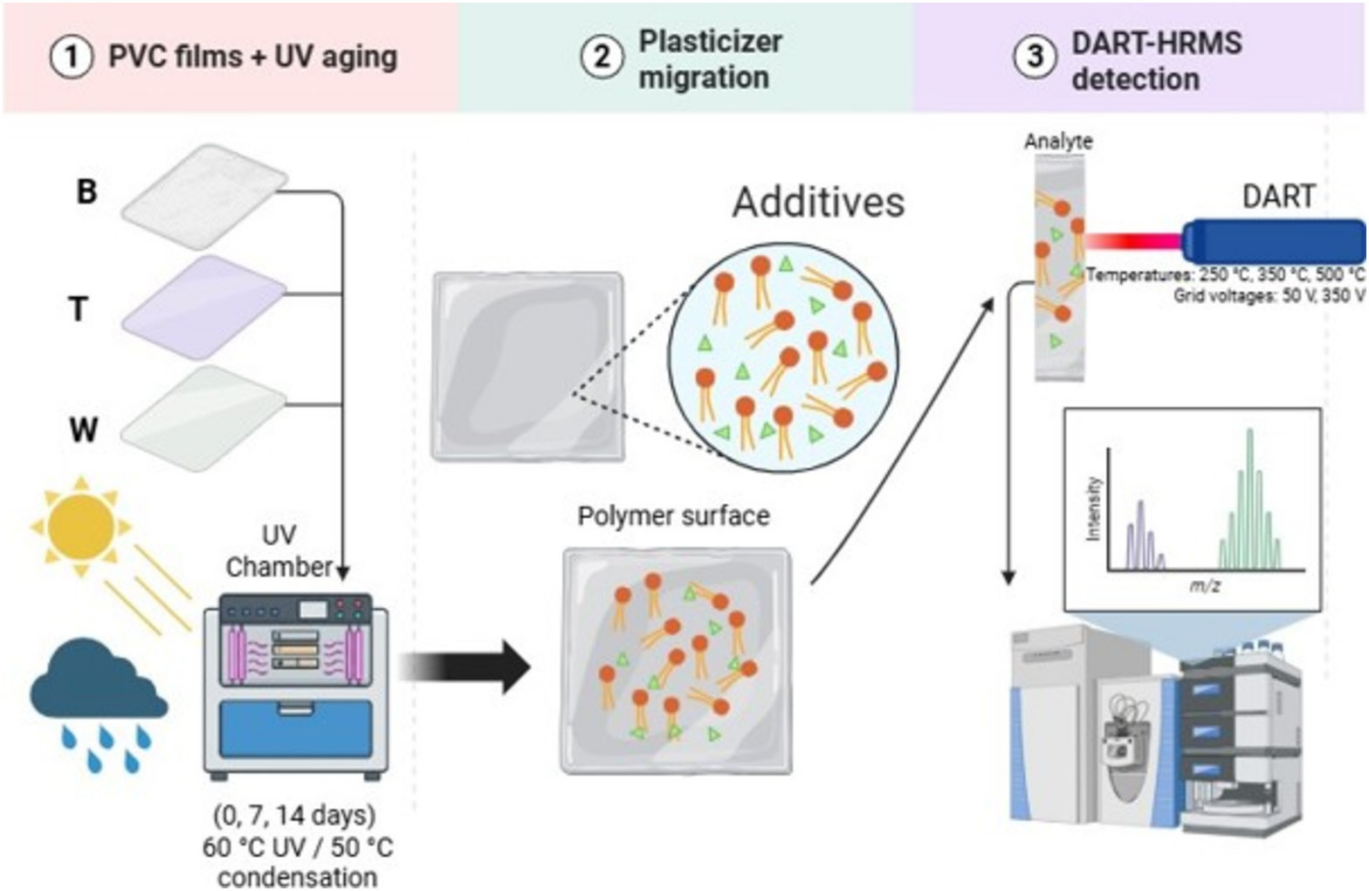
Schematic representation of the workflow for UV aging of PVC films, plasticizer migration to the surface, and direct detection by DART‐HRMS.

In this study, we evaluated commercial PVC films subjected to UV‐controlled aging to establish an optimized analytical approach based on DART‐HRMS for the rapid screening and monitoring of plasticizer migration (Figure 1).

By correlating variations in ionic signal intensity with UV and heat‐induced progressive degradation, the method seeks to elucidate the relationship between the evolution of surface chemistry and the desorption behavior of the additive. This approach provides a high‐throughput alternative for evaluating the stability and release potential of phthalate plasticizers in polymeric materials, thereby improving analytical reliability and the safety assessment of consumer‐grade PVC products.

## 2 Materials and Methods

### 2.1 Materials and PVC Films

Three commercially available flexible PVC films from local suppliers were selected for this study. To preserve manufacturers' anonymity and focus exclusively on analytical performance, the films were coded as B, T, and W. All materials were transparent or translucent, plasticized PVC with a similar nominal thickness (0.15–0.25 mm), as commonly used in packaging, school supplies, and household goods. The manufacturers provided no prior information on formulation, additives, or stabilizer content.

### 2.2 Accelerated UV Aging Protocol

Accelerated aging of PVC films was performed in a UV fluorescence apparatus in accordance with ASTM G154‐23 [26]. Samples were exposed to alternating cycles of 8 h of UV irradiation at 60 °C followed by 4 h of condensation at 50 °C, using UVA‐340 lamps with an irradiance of 0.89 W/(m^2^ × nm) at 340 nm (Cycle A). This protocol resulted in a total cumulative UV dose of approximately 359 kJ/m^2^ for 7 days and 718 kJ/m^2^ for 14 days of exposure.

For each commercial PVC film (Codes B, T, and W), one set of samples was kept unexposed and served as the 0‐day condition (B1, T1, and W1). The remaining sets were placed in the UV chamber and subjected to 7 (B2, T2, and W2) and 14 days (B3, T3, and W3) of continuous aging under the programmed cycle. After aging, all samples were stored in desiccators at room temperature to minimize loss through volatilization or additional light exposure before DART‐HRMS analysis.

### 2.3 DART‐HRMS Instrumentation and Acquisition Conditions

High‐resolution mass spectra were acquired using a Q Exactive Orbitrap mass spectrometer (Thermo Fisher Scientific, Bremen, Germany) coupled to an IonSense DART JumpShot ion source (Saugus, MA, USA), operated in positive ion mode. Helium (He, 99.999%) was used as both the ionizing and carrier gas. The distance between the DART outlet and the MS inlet was fixed at 3 cm. To ensure reproducible sample positioning and address potential surface heterogeneity, all films were introduced into the ionization region using an automated sampling stage at 1 mm/s, allowing for a representative longitudinal scan of the surface. The DART needle potential was set to 300 V, while the grid potential was varied between 50 and 350 V. The ionization gas temperature was adjusted to 250 °C, 350 °C, or 500 °C, according to the experimental design. All PVC films were analyzed directly, without solvent extraction or chemical pretreatment, and were placed in a flat position on the DART sampling rods (QuickStrip holders). Each film was analyzed in triplicate, with measurements taken across different surface regions to minimize variations in ionic transmission and ensure the statistical representativeness of the migration profiles.

Mass spectra were collected in full‐scan mode from *m/z* 70 to *m/z* 1050, at a resolving power of 70 000 (FWHM at *m/z* 200), with an AGC target of 1 × 10^6^ and a maximum injection time of 100 ms. External calibration was performed daily using the manufacturer's standard solution to maintain sub‐ppm mass accuracy. To systematically evaluate the effects of desorption temperature and ion transmission energy, the analyses were performed using the following DART condition matrix (Table 1). Each film was analyzed in triplicate under every DART condition to ensure reproducibility.

**TABLE 1:** DART‐HRMS experimental conditions used.

No.	Temperature (°C)	Grid voltage (V)
1	250	50
2	350	50
3	500	50
4	250	350
5	350	350
6	500	350

### 2.4 Target Compounds and Ion Monitoring

The monitored species included diethyl phthalate (DEP), as well as DBP and its structural isomer, diisobutyl phthalate (DIBP). Because the latter two have identical mass‐to‐charge ratios, they are reported together as DBP/DIBP. Similarly, the C_8_ phthalate/terephthalate isomeric series, comprising DEHP, di‐*n*‐octyl phthalate (DOP), diisooctyl phthalate (DIOP), and dioctyl terephthalate (DOTP), cannot be differentiated in full‐scan mode. Therefore, these species are collectively defined here as the “DEHP group.” The method also targeted higher molecular weight phthalates (DINP and DIDP) and dimethyl sulfoxide (DMSO) as process residue. A summary of the target analytes is presented in Table 2.

**TABLE 2:** Target compounds monitored.

No.	Compound	Molecular formula	Theoretical *m/z* [M + H]^+^	Structural formula
1	Diethyl phthalate (DEP)	C_12_H_14_O_4_	223.0965	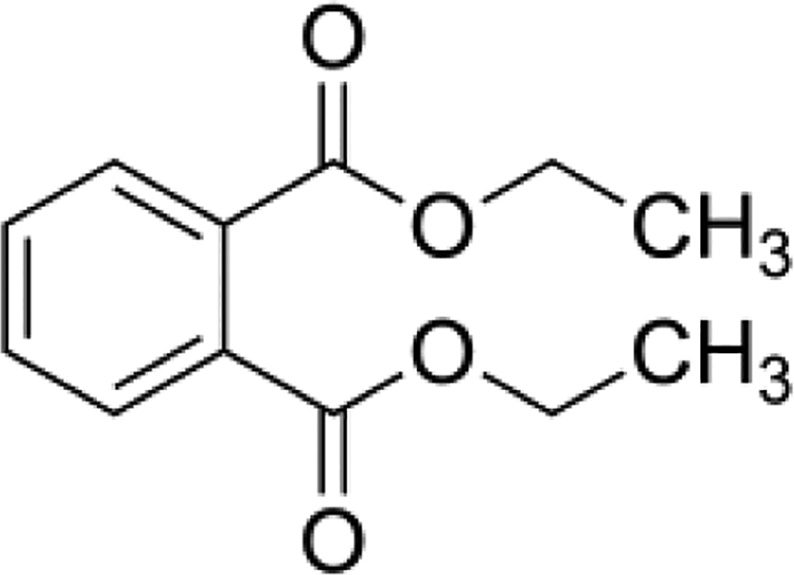
2	Dibutyl/diisobutyl phthalate (DBP/DIBP)^a^	C_16_H_22_O_4_	279.1591	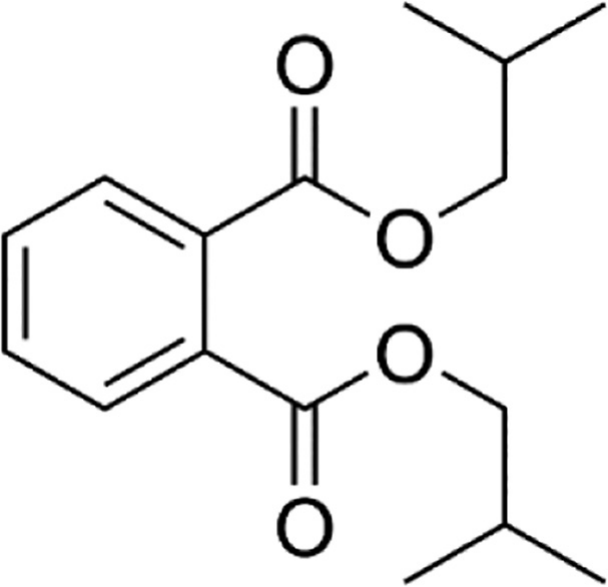
3	C_8_ phthalate/terephthalate group: DEHP/DOP/DIOP/DOTP^b^	C_24_H_38_O_4_	391.2853	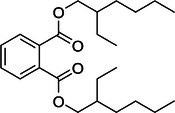
4	Diisononyl phthalate (DINP)	C_26_H_42_O_4_	419.3166	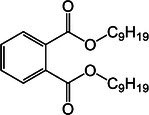
5	Diisodecyl phthalate (DIDP)	C_28_H_46_O_4_	447.3479	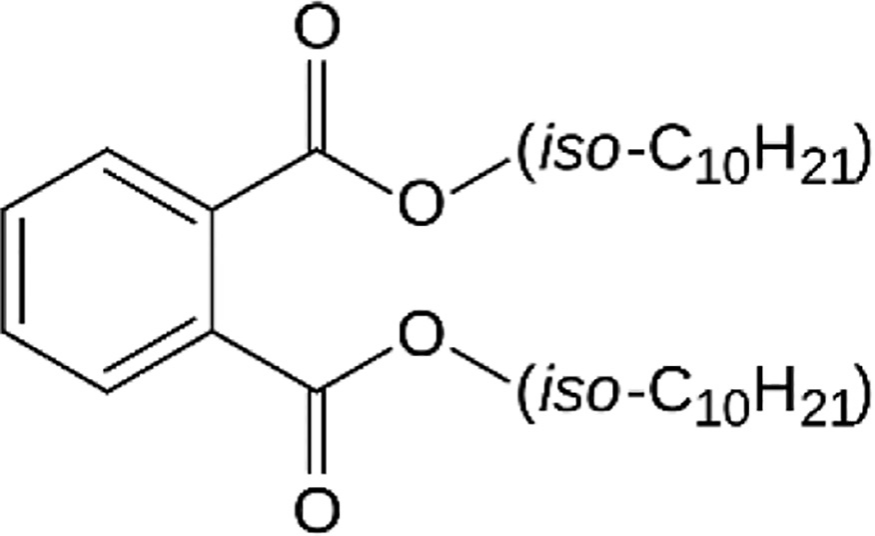
6	Dimethyl sulfoxide (DMSO)	C_2_H_6_OS	79.0212	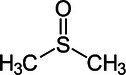

^a^ DBP and DIBP are structural isomers; the structure shown represents the DBP backbone.

^b^ DEHP, DOP, DIOP, and DOTP share the same elemental composition (C_24_H_38_O_4_). The structure shown represents the DEHP core, used as a generic model for the C_8_ phthalate/terephthalate group. Under full‐scan DART‐HRMS conditions, these isomers produce identical [M + H]^+^ ions and cannot be differentiated.

### 2.5 Thermogravimetric Analysis (TGA)

TGA was performed using a Shimadzu TGA‐50 analyzer to evaluate the thermal stability of the PVC films. Samples of 4–10 mg were placed in platinum pans and heated from 25°C to 600°C at a heating rate of 10°C/min under a nitrogen atmosphere at 60 mL/min.

### 2.6 Data‐Processing Analysis

Data processing was performed using Xcalibur 3.2 software (Thermo Fisher Scientific, MA, USA) with a mass tolerance of ±10 ppm. Signal intensities were reported as the mean of three replicates. The influence of DART parameters was evaluated using a two‐way ANOVA followed by a heteroscedastic two‐tailed Student's *t* test (*p* < 0.05) to identify optimal conditions. Statistical analysis was carried out in Microsoft Excel, and graphs were plotted using OriginPro (OriginLab, Northampton, MA, USA).

## 3 Results and Discussion

### 3.1 Effect of DART Conditions on Plasticizer Signal and Integrity

**FIGURE 2:**
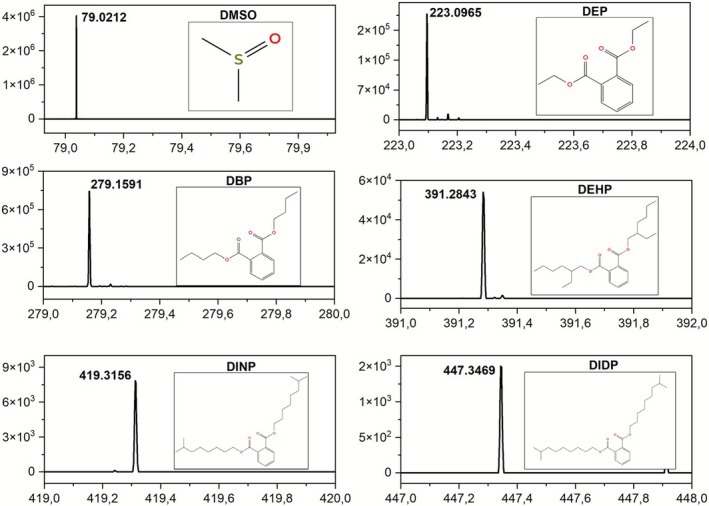
Identification of phthalate plasticizers and DMSO in commercial PVC films by DART‐HRMS using an ionization gas temperature of 350°C and a grid voltage of 50 V.

Nonaged PVC films analysis revealed a consistent additive profile across all brands, confirming the presence of plasticizers and solvent residues, and although absolute signal intensities varied depending on the specific commercial formulation and instrumental parameters, the qualitative profile remained consistent across all nonaged samples (B1, T1, and W1). Table S1 presents the detailed mass spectrometry parameters for DEP as a representative dataset, and this high instrumental accuracy was consistent across the method, and all monitored additives were confirmed with a mass error below 10 ppm. Due to the extensive volume of experimental data generated, complete tables for the remaining compounds and full statistical datasets are available from the corresponding author upon request. Figure 2 displays representative mass spectra of the fresh PVC film (Brand B) acquired at an ionization gas temperature of 350°C and a grid voltage of 50 V.

Gas temperature plays a dual role, providing energy for the thermal desorption of analytes and for their ionization. Adjusting this parameter requires balance; excessive heat can degrade the ion of interest, while insufficient levels fail to induce ionization. Simultaneously, the grid voltage is fundamental for avoiding ionic recombination, directing the flow to the mass spectrometer, and enabling Penning ionization [27, 28]. Therefore, the influence of these variables was systematically evaluated to ensure efficient volatilization of additives in PVC while preserving ion stability. It was observed that both parameters strongly impact the detection of phthalates and residues, a sensitivity exacerbated by the thin film geometry, in which slight variations in positioning alter ionic transmission.

TGA was employed as a complementary tool to provide qualitative confirmation of the inherent thermal stability of the PVC samples. Figure S1 shows that the TGA curves exhibit a primary decomposition onset temperature (Tonset) in the range of approximately 240°C to 300°C. This temperature range is consistent with the onset of PVC dehydrochlorination, widely recognized as the main stage of the polymer's thermal degradation, which typically occurs between 200°C and 360°C [28, 29]. Higher *T*_onset_ values are expected for commercially formulated PVC due to the presence of plasticizers, stabilizers, and fillers [30]. Despite the onset of matrix degradation within this temperature range, DART‐HRMS experiments demonstrated that gas temperatures between 250°C and 350°C remain suitable for surface desorption of additives, preserving the molecular integrity of plasticizers and enabling their reliable detection.

**FIGURE 3:**
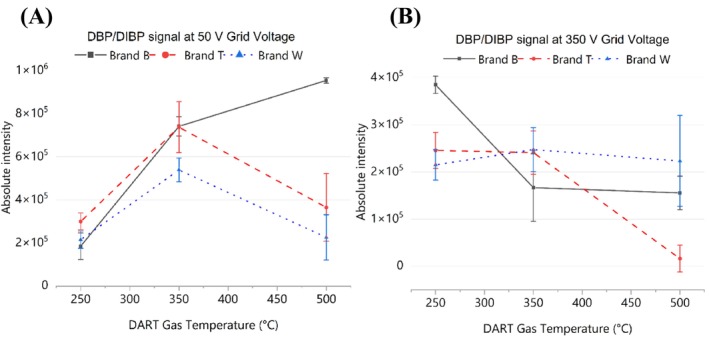
Effect of DART gas temperature on the absolute intensity of *m/z* 279.1591 [M + H]^+^ (DBP/DIBP). (A) Grid voltage of 50 V. (B) Grid voltage of 350 V. Error bars indicate standard deviation (*n* = 3).

The evaluation of short‐chain phthalates (DEP and DBP/DIBP) demonstrated that ionization optimization critically depends on grid voltage modulation. These observations were corroborated by statistical analysis, as exemplified by the DBP/DIBP results presented in Table S2. Complete statistical datasets for the remaining analytes are available from the corresponding author upon request. Using DBP/DIBP as a representative model (Figure 3), a general thermal trend was observed in the low‐voltage regime (Figure 3A), where signal intensities typically increased from 250°C to 350°C due to enhanced desorption. However, signals decreased at 500°C, suggesting thermal degradation. Notably, this behavior was not universal; in some cases, intensities remained stable or continued to increase at 500°C, indicating that susceptibility to thermal degradation is matrix‐dependent and varies according to the specific PVC formulation. Conversely, at high grid voltages (Figure 3B), a two‐way ANOVA revealed that voltage became the most influential factor for almost all analytes (*p* < 0.001), consistently reducing signal intensities and increasing variance compared with the 50‐V condition.

Temperature also had a significant main effect (*p* < 0.05), but its magnitude strongly depended on the applied voltage. This dependence was confirmed by the critical interaction between voltage and temperature (*p* < 0.05), demonstrating that the gain in thermal intensity occurred only in the moderate extraction regime (50 V). Paired *t* tests corroborated this conclusion; at 50 V, the contrast between 250°C and 350°C was statistically significant (*p* < 0.05) for both DEP and DBP/DIBP, confirming an ideal thermal window. In sharp contrast, at 350 V, temperature comparisons were largely nonsignificant (*p* > 0.05), indicating that excess kinetic energy in the source neutralizes the benefits of thermal desorption, resulting in signal suppression and instability, which is consistent with voltage‐induced fragmentation and unstable desorption rates.

Medium‐chain phthalates (DEHP/DOP/DIOP group) exhibited intermediate behavior during optimization. Although consistently detected, their response to DART was strongly influenced by the parameter combination, showing a sharp intensity peak at 350°C. Unlike short‐chain phthalates, for which the signal increase from 250°C to 350°C was statistically significant (*p* < 0.05) under the low‐voltage condition (50 V), the thermal gain for the DEHP group was less robust at 50 V (approximately *p* = 0.06 for some matrices). This suggests a higher volatilization inertia, requiring greater energy input. However, two‐way analysis of variance (ANOVA) revealed a highly significant interaction between temperature and voltage (with *p* values as low as 0.0007), indicating that molecular stability is the determining factor in voltage selection. At 50 V, DEHP maintained a controllable desorption profile. In stark contrast, at 350 V, a sharp and statistically significant decline in signal intensity (*p* < 0.01) was observed at 500°C, leading to near‐total signal suppression for some brands. This phenomenon is consistent with voltage‐induced fragmentation and confirms that, despite the less significant thermal gain, the 50‐V condition is indispensable for preserving the molecular integrity of the DEHP group. It is important to note that while the current full‐scan approach does not differentiate between DEHP, DOP, DIOP, and DOTP isomers, the preservation of the molecular ion [M + H]^+^ at 50 V is a prerequisite for future identification steps. As demonstrated by Kuki et al. [21], these isomers can be effectively distinguished by DART‐MS/MS through the analysis of specific fragment intensity ratios (such as alkene vs. fatty alcohol losses), provided that the collision energy is appropriately tuned.

Despite these variations, the impact of high voltage was critical for heavier plasticizers. As shown in Table 3, DINP and DIDP signals decreased significantly, in some cases falling below the detection limit at 350 V. For these high‐molecular‐weight species (C_9_–C_10_), the absence of statistical significance in the ANOVA results was generally observed, which is attributed to the extremely low absolute signal intensity (typically < 1000 counts), frequently operating near the quantification limit of the method. This indicates that the low vapor pressure and low concentration in the matrix limit efficient thermal desorption, while the high kinetic energy at 350 V suppresses the remaining signal, confirming that the 50‐V condition is indispensable for their detection. Finally, for DMSO (*m/z* 79), ANOVA confirmed that grid voltage was the most critical factor (*p* < 0.001), with the 50‐V condition producing signals that were, on average, an order of magnitude (approximately 10–20 times) higher than those obtained at 350 V. This effect indicates that high grid potentials severely hinder ion transmission. Although the main effect of temperature was not statistically significant for Brands B and W (*p* > 0.05) due to high volatility and variance, the significant interaction (Voltage × Temperature) confirmed that the positive thermal gain at 350°C occurs only in the milder 50‐V regime.

**TABLE 3:** Intensities (raw values) of all monitored additives at 50 and 350 V.

Compound	[M + H]+	Brand B			Brand T			Brand W		
		250°C	350°C	500°C	250°C	350°C	500°C	250°C	350°C	500°C
GRID 50 V										
DEP	223.0965	5.2E+04	2.6E+05	1.8E+05	1.1E+05	2.1E+05	6.4E+04	4.5E+04	1.4E+05	1.1E+05
DBP/DIBP	279.1591	1.9E+05	7.4E+05	9.5E+05	3.0E+05	7.4E+05	3.7E+05	2.2E+05	5.4E+05	2.3E+05
DEHP group	391.2853	1.2E+04	4.9E+04	1.1E+05	1.3E+04	5.5E+04	3.3E+04	9.5E+03	3.7E+04	3.5E+04
DINP	419.3166	1.2E+03	4.7E+03	3.0E+03	8.0E+02	2.8E+03	1.9E+03	1.3E+03	1.0E+03	1.1E+03
DIDP	447.3479	2.6E+02	8.5E+02	5.7E+02	3.9E+01	8.0E+02	3.6E+02	2.3E+02	1.7E+02	1.6E+02
DMSO	79.0212	1.2E+06	2.8E+06	2.6E+06	1.1E+06	3.9E+06	2.5E+06	1.2E+06	2.3E+06	2.1E+06
GRID 350 V										
DEP	223.0965	2.8E+04	1.9E+04	2.8E+04	3.9E+04	2.7E+04	4.9E+02	2.6E+04	2.5E+04	2.9E+04
DBP/DIBP	279.1591	3.8E+05	1.7E+05	1.6E+05	2.5E+05	2.4E+05	1.6E+04	2.2E+05	2.5E+05	2.2E+05
DEHP group	391.2853	4.1E+04	6.7E+04	7.6E+03	2.2E+04	4.2E+04	6.2E+03	1.6E+04	7.9E+04	3.4E+04
DINP	419.3166	1.6E+03	1.3E+03	1.7E+02	5.0E+02	7.4E+02	ND	5.0E+02	1.3E+03	1.7E+03
DIDP	447.3479	4.1E+02	4.4E+02	ND	2.1E+01	1.4E+02	ND	4.1E+01	2.4E+02	1.8E+02
DMSO	79.0212	7.5E+05	4.3E+04	1.5E+05	2.3E+05	1.5E+05	7.0E+03	1.7E+05	2.9E+05	1.3E+05

Based on the values shown in Table 3 and the statistical results, the 350 °C/50 V configuration was chosen because the low grid voltage (50 V) was the most critical factor (*p* < 0.001), proving indispensable for preserving molecular integrity and avoiding severe fragmentation and signal suppression caused by 350 V in most samples. The temperature of 350°C provided the maximum thermal desorption efficiency, a gain that proved effective only when combined with the moderate 50‐V condition (significant interaction).

DBP/DIBP (*m/z* 279.1591) was identified as the primary plasticizer, exhibiting significantly higher signal intensities than secondary plasticizers such as DEP (*m/z* 223.0965) and the DEHP group (*m/z* 391.2853). As the present study was designed as a high‐throughput qualitative screening approach, higher molecular‐weight phthalates (DINP and DIDP) were detected only at trace levels, suggesting minimal incorporation into the original formulations. Notably, DMSO (*m/z* 79.0212) appeared as the base peak in several spectra, particularly for Brand T, indicating substantial solvent retention from the manufacturing process within the polymer matrix. This detection capability is consistent with the parameters established by Rothenbacher and Schwack [19], who reported a detection limit of approximately 1% (w/w) for DBP and DEHP in PVC plastisols using direct DART screening. Similarly, Kuki et al. [21] successfully employed DART‐HRMS to rapidly identify several phthalates, including DOP, DDP, DEHP, and DINP, in consumer PVC films.

### 3.2 Migration Behavior During UV Aging at 0, 7, and 14 Days

**FIGURE 4:**
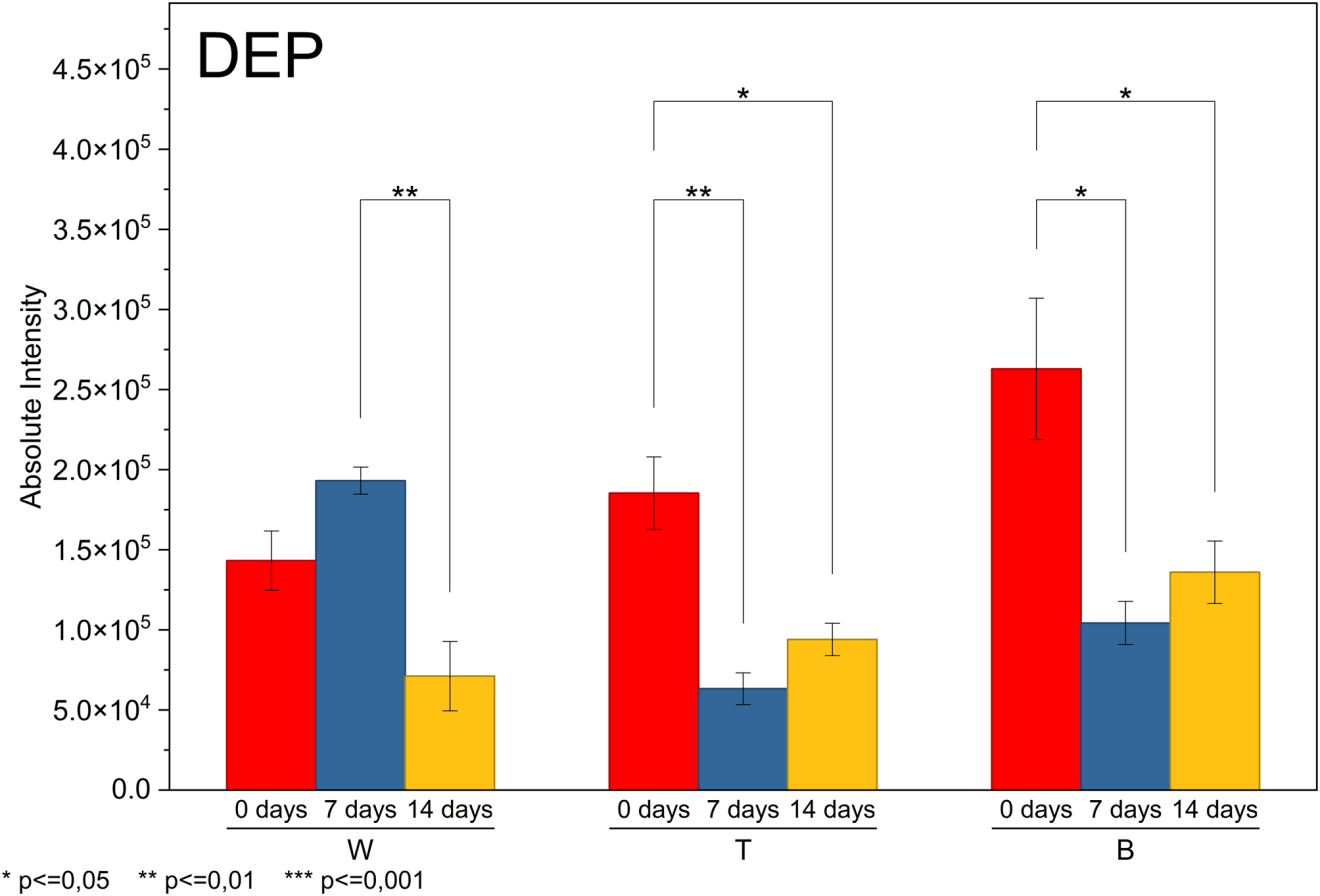
Surface migration profile of DEP.

The migration behavior of DEP and DBP/DIBP during UV aging revealed clear brand‐dependent dynamics that reflect differences in polymer microstructure and additive retention. The complete statistical evaluation supporting these trends, including ANOVA and *t* test results for all monitored additives, is detailed in Table S3. For DEP, all three brands showed significant temporal effects according to one‐way ANOVA, confirming that UV exposure altered the surface availability of this highly mobile short‐chain phthalate, as shown in Figure 4. In Brand W, the average intensity increased from 0 to 7 days, but not significant according to the *t* test (*p* = 0.10), indicating that migration had started, but had not yet reached a statistically detectable limit. Between 7 and 14 days, however, DEP intensity dropped dramatically, and the difference became significant (*p* = 0.02), revealing that after initial external diffusion, prolonged exposure to UV rays led to volatilization or photodegradation, consistent with the known high mobility of DEP [29]. Brand T exhibited the opposite initial pattern, DEP decreased significantly from 0 to 7 days (*p* = 0.02), suggesting that this formulation either retains DEP more effectively, delaying surface enrichment, or loses DEP quickly during the first few UV cycles. Although the increase from 7 to 14 days was not statistically significant (*p* = 0.10), the observed rebound suggests that cumulative structural damage over time, such as dehydrochlorination, microcracking, and increased porosity, enabled secondary migration, indicating a delayed but eventual release of DEP. For Brand B, neither the decrease from 0 to 7 days nor the subsequent increase at 14 days was statistically significant (*p* > 0.05), indicating a more gradual migration behavior that remained below the threshold of statistical detectability throughout the evaluated period. This moderate response likely reflects a formulation with higher retention capacity, the presence of stabilizers that limit additive mobility, or differences in sample positioning during DART analysis.

**FIGURE 5:**
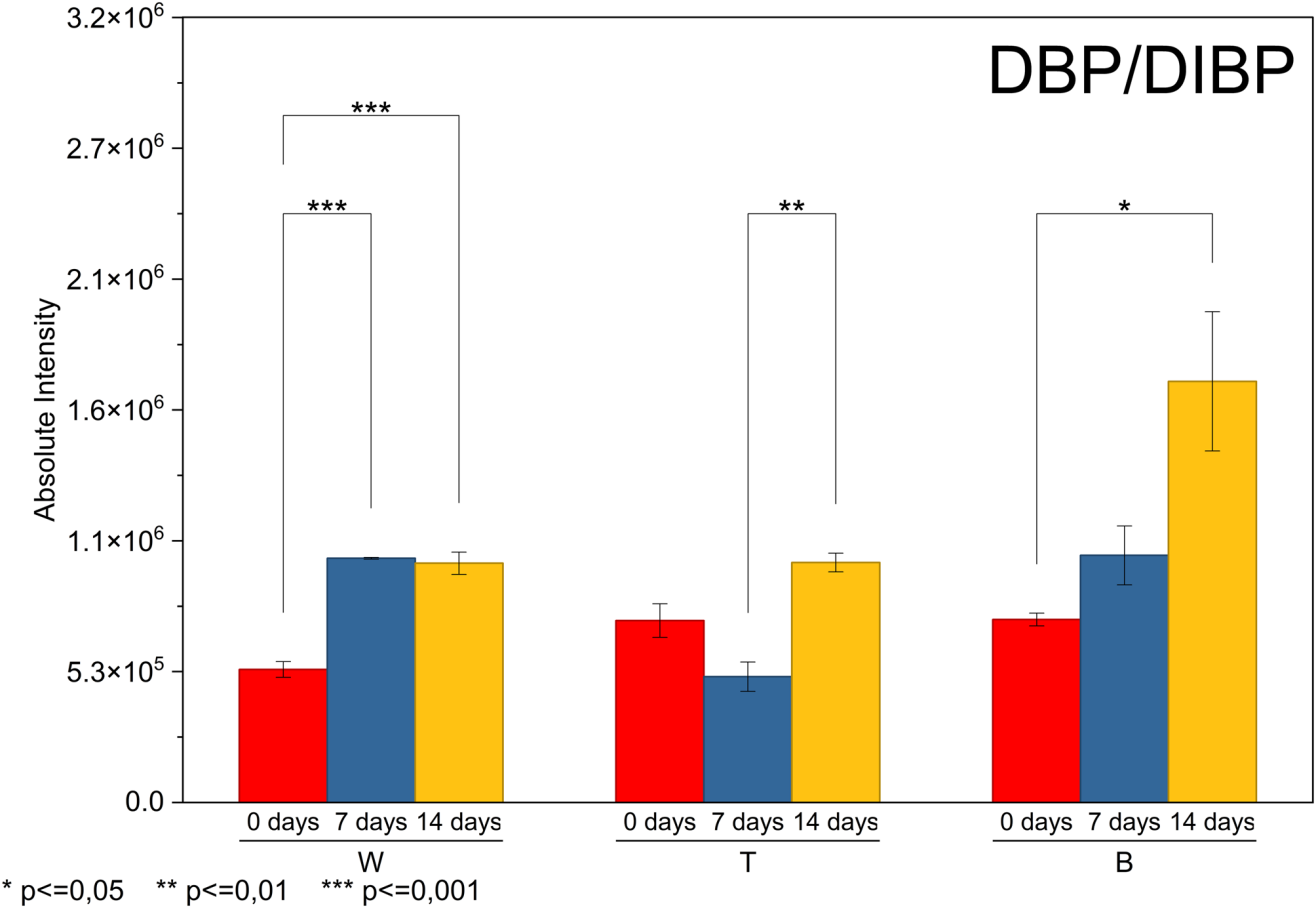
Surface migration profile of DBP/DIBP.

As illustrated in Figure 5, the behavior of DBP/DIBP, a short‐chain phthalate with lower volatility, was markedly different but still strongly influenced by UV exposure, as confirmed by the significant ANOVA results for all brands. At Brand W, DBP/DIBP exhibited a clear migration event, the intensity from 0 to 7 days increased markedly and significantly (*p* = 0.005), confirming a robust surface enrichment driven by UV‐induced relaxation and increased free volume. Comparison from 7 to 14 days showed no significant change (*p* = 0.70), while 0–14 days remained significant (*p* = 0.002), indicating that by 7 days, the migration plateau was essentially reached, with little further release thereafter. The T brand presented a more complex profile: The decrease from 0 to 7 days was not significant (*p* = 0.07), meaning that migration had not yet manifested itself strongly on the surface; however, the increase from 7 to 14 days was significant (*p* = 0.005), demonstrating delayed migration, consistent with a matrix that initially restricts diffusion but becomes more permeable after prolonged photodegradation. At Brand B, none of the pairwise comparisons reached significance (*p* > 0.05), despite visible increases in signal, suggesting that although PAD/DIBP migrates, the process is gradual and dampened by the formulation, remaining below the statistical threshold within the period evaluated.

**FIGURE 6:**
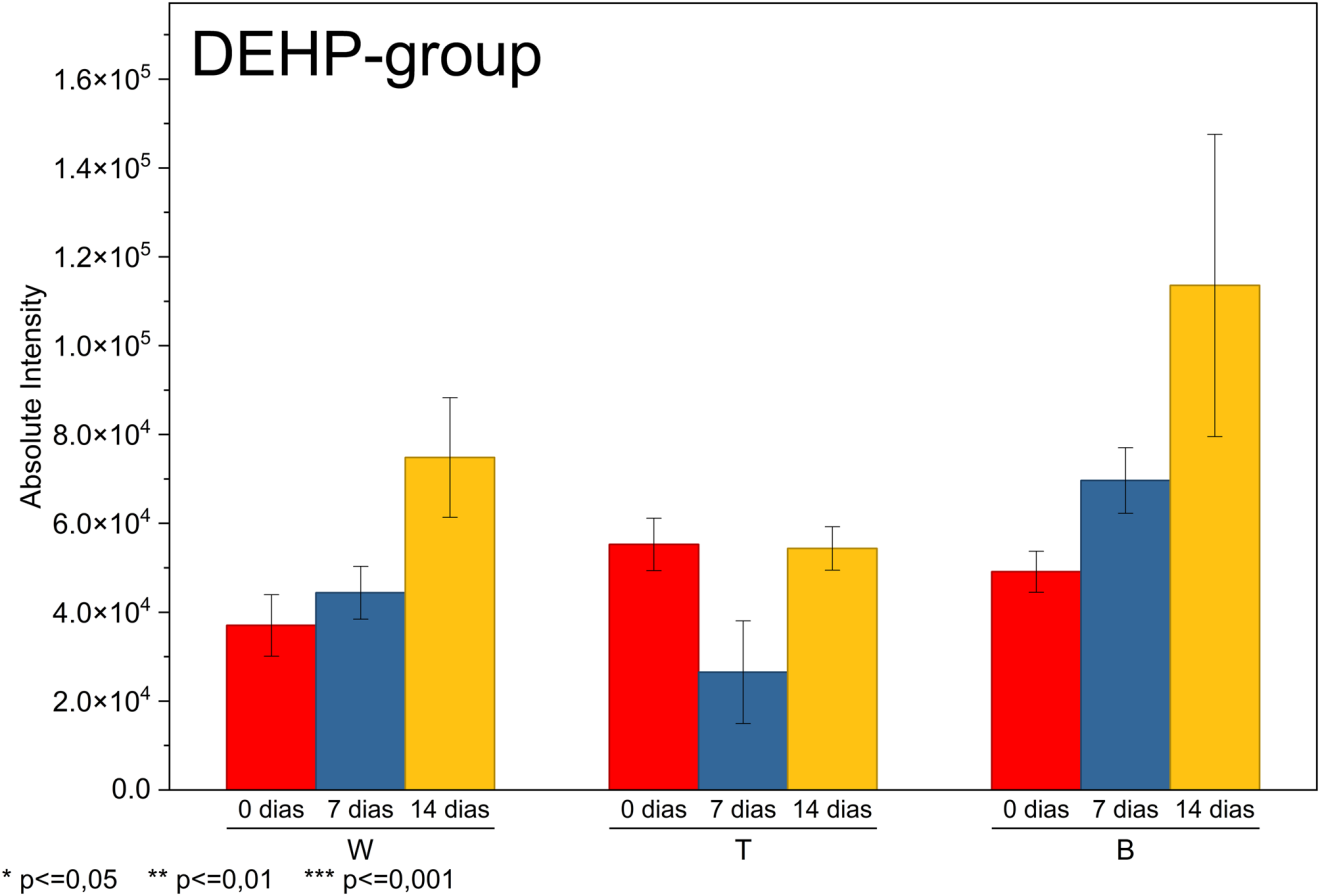
Surface migration profile of DEHP group.

The behavior of the DEHP group during UV aging, as shown in Figure 6, was markedly different from that observed for the more volatile plasticizers (DEP and DBP), revealing considerably lower mobility and, in some cases, an absence of detectable migration in the first 2 weeks of exposure. Two‐way ANOVA revealed no significant interaction between the brand and exposure time (*p* > 0.05), nor was there a significant main effect for time. Although some apparent increases in the means were observed, especially at 14 days, these variations remained within the experimental range and did not constitute effective migration. These results may be associated with lower volatility, higher molecular weight, and the strong affinity of the DEHP group for the PVC matrix, factors that confer greater resistance to surface diffusion and delay mobility even under intense photodegradation conditions. The low mobility of DEHP is well documented, as shown by Russell and McDuffie [30], it can be attributed to its high partition coefficient, which indicates a strong tendency to sorb in the solid phase, in contrast to the high or moderate mobility observed for DEP and DBP.

**FIGURE 7:**
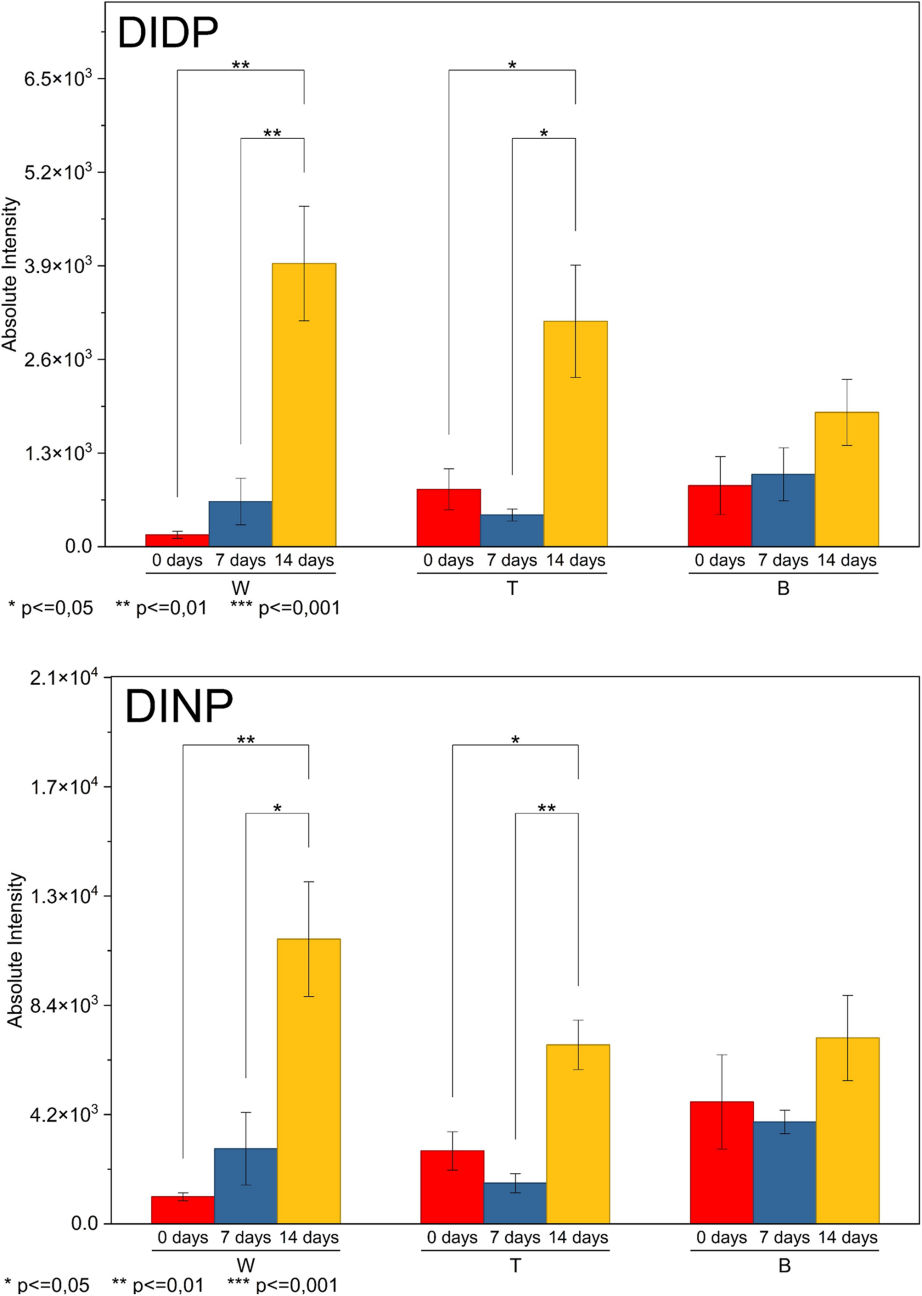
Surface migration profile of DIDP and DINP.

As shown in Figure 7, the plasticizers with higher molar mass (DINP and DIDP) exhibited a migration pattern characterized by increased signal and the onset of migration after 14 days, only in brands W and T. For both plasticizers, ANOVA indicated significant overall differences in Brands W and T, for both DINP and DIDP, while Brand B did not show statistically relevant variations during aging (*p* > 0.05). This observation suggests that the mobility of these plasticizers is dependent on the specific characteristics of the PVC formulation.

In the *t* tests, a consistent pattern was observed where the 0‐ versus 7‐day comparison showed no differences (*p* > 0.20 across all brands). This suggests that no detectable migration occurred during the first week of UV exposure. However, the 7‐ versus 14‐day comparison showed significant differences for both DINP and DIDP in Brand W, indicating that migration only becomes evident after 2 weeks. Comparing 0 versus 14 days also yielded significance only for Brand W, which may be associated with a slow and progressive migration behavior, consistent with the low mobility of these plasticizers [31]. Collectively, DINP and DIDP demonstrate low initial mobility, an absence of measurable migration until 7 days, and a marked, statistically significant increase only after 14 days, which was limited to Brand W and partially observed in Brand T. This behavior is consistent with their high molar masses and greater affinity for the polymer matrix, which retards surface diffusion. For Brand B, the absence of significance suggests more effective diffusional barriers or greater internal retention throughout the 2 weeks evaluated.

**FIGURE 8:**
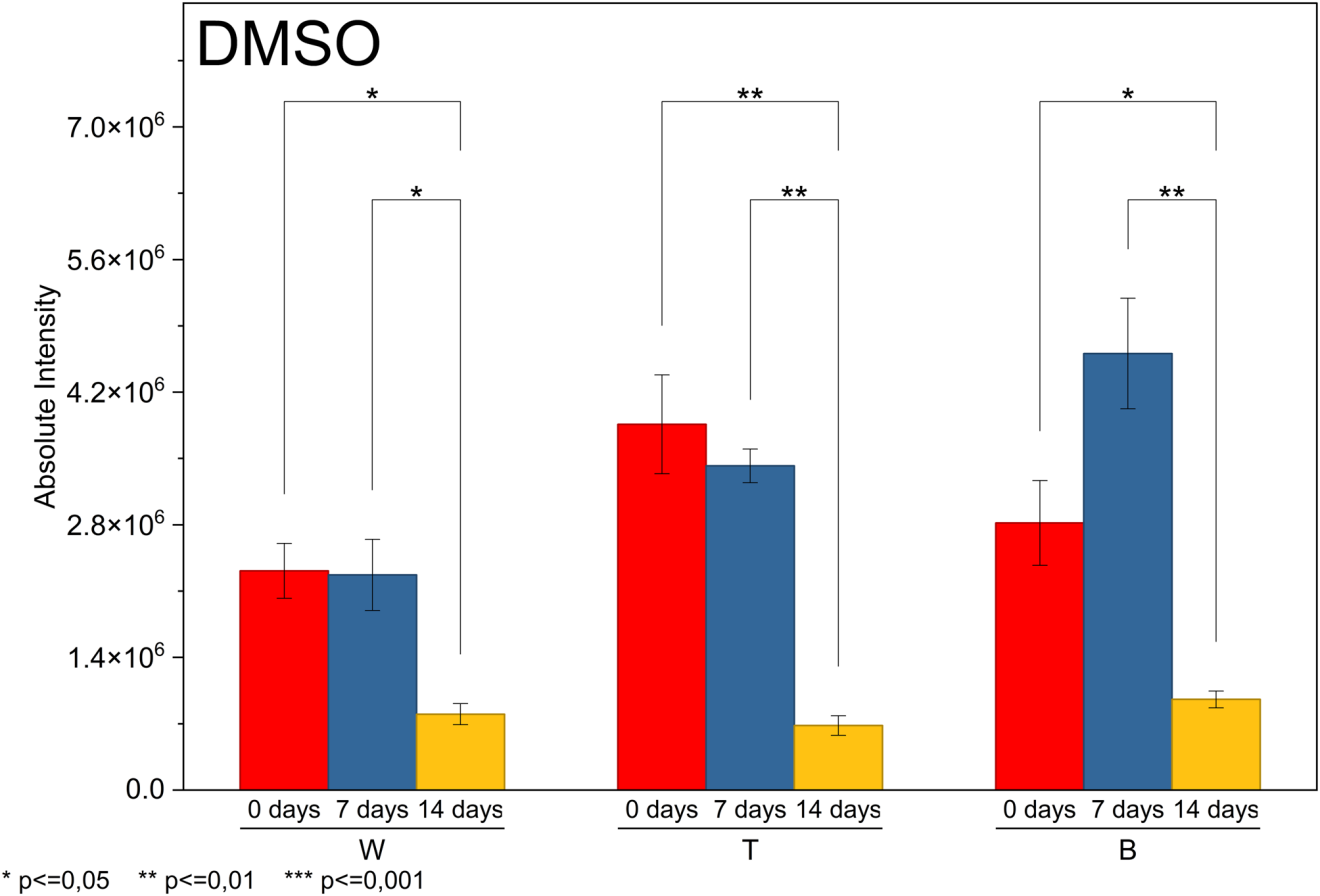
Surface migration profile of DMSO.

The behavior of DMSO is illustrated in Figure 8, revealing a sharp decline pattern between 0 and 14 days, characterizing a significant surface loss throughout the exposure. ANOVA indicated significant overall differences between time points (*p* < 0.05) in all brands, confirming that the observed variation is not merely due to experimental variability.

In *t* tests, the contrast between Days 0 and 7 showed no significance in any brand (*p* > 0.05), indicating that migration was not yet clearly detectable in the first 7 days. However, the 7‐ versus 14‐day interval showed significance for all brands (*p* ≤ 0.05), providing evidence that most DMSO loss/migration occurs after the first week, at a point where the PVC is already significantly degraded by photodegradation (dechlorination, polyene formation, and surface cracking). The contrast between 0 and 14 days was also significant (*p* ≤ 0.05) in all three brands, consolidating the interpretation that DMSO undergoes late, but intense, migration. This is typical of a low molar mass solvent that initially remains retained but volatilizes rapidly after structural modifications induced by UV exposure. Thus, DMSO exhibits low resistance to migration, but the process itself is not immediate, depending on the chemical and morphological changes accumulated in the polymer [32].

## 4 Conclusion

This study demonstrates that DART‐HRMS is a powerful and efficient tool for the rapid, extraction‐free monitoring of plasticizer migration in PVC films subjected to UV‐induced aging. Systematic optimization of ionization gas temperature and grid voltage showed that analytical performance is sensitive to source energy, with the 350 °C/50 V configuration providing the optimal balance between thermal desorption efficiency and molecular integrity. Under these conditions, both low‐ and high‐molecular‐weight additives could be reliably detected, while avoiding the extensive fragmentation and signal suppression observed at elevated grid voltages.

Application of the optimized method to aged commercial PVC films revealed clear, formulation‐dependent migration profiles. Short‐chain phthalates exhibited rapid surface enrichment, followed in some cases by signal decline consistent with volatilization or photodegradation. In contrast, medium‐ and high‐molecular‐weight plasticizers showed delayed and limited migration, becoming detectable only after prolonged UV exposure, reflecting their lower volatility and stronger affinity for the polymer matrix. The detection of DMSO further highlighted DART‐HRMS's ability to monitor process‐related residues and capture late‐stage release phenomena associated with advanced polymer degradation.

Overall, the results confirm that the combined effects of molecular size, volatility, formulation characteristics, and UV‐induced structural changes such as dehydrochlorination and increased free volume govern additive mobility in PVC. By enabling rapid, surface‐specific chemical screening without sample preparation, DART‐HRMS emerges as a valuable high‐throughput tool for studying additive migration, supporting regulatory compliance, materials safety assessment, and the development of more stable and safer PVC formulations. Future studies should evaluate how different PVC thicknesses, fillers, and stabilizers affect migration kinetics. Expanding this DART‐HRMS approach to a broader range of formulations will further validate its generalizability for safety assessments.

## Author Contributions

**Odilon Leite‐Barbosa:** methodology, investigation, data curation, formal analysis, visualization, writing – original draft. **Marcelo Ferreira Leão de Oliveira:** methodology, validation, resources, writing – review and editing. **Márcia Gomes de Oliveira:** conceptualization, resources, supervision, writing – review and editing. **Monica Costa Padilha:** methodology, resources, validation, writing – review and editing. **Valdir Florêncio Veiga‐Junior:** conceptualization, project administration, funding acquisition, supervision, writing – review and editing.

## Funding

This work was supported by the Carlos Chagas Filho Foundation for Research Support of the State of Rio de Janeiro (FAPERJ) (Grant Numbers E‐26/211.315/2021 and E‐26/200.512/2023) and the National Council for Scientific and Technological Development (CNPq) (Grant Number 310782/2022‐8).

## Conflicts of Interest

The authors declare no conflicts of interest.

## Supporting information


**Table S1:** Detailed DART‐HRMS data for diethyl phthalate (DEP) (C_12_H_14_O_4_; theoretical *m/z* [M + H]+: 223.0965).
**Table S2:** Statistical optimization analysis (two‐way ANOVA and Student's *t* tests) for dibutyl phthalate (DBP/DIBP) signal intensity across three PVC brands (0 days).
**Table S3:** Comprehensive statistical evaluation of migration trends (one‐way ANOVA and Student's *t* tests) for all monitored additives in PVC films during UV aging (0, 7, and 14 days).
**Figure S1:** Tg analysis curves for the three commercial PVC films subjected to UV aging: (A) Brand B, (B) Brand T, and (C) Brand W. The curves display the thermal degradation profiles for unexposed samples (0 days) compared with those aged for 7 and 14 days.

## Data Availability

The data that support the findings of this study are available from the corresponding author upon reasonable request.
