# Supplementary material for: Rapid Detection of Plasticizer Migration From UV‐Aged PVC Films by DART‐HRMS
Source: Rapid Commun Mass Spectrom. 2026 Feb 17;40(10):e70048. doi: 10.1002/rcm.70048 (PMC12911472; doi:10.1002/rcm.70048)
Supplement: Supplementary file 1 — Table S1: Detailed DART‐HRMS data for diethyl phthalate (DEP) (C12H14O4; theoretical m/z [M + H]+: 223.0965). Table S2: Statistical optimization analysis (two‐way ANOVA and Student's t tests) for dibutyl phthalate (DBP/DIBP) signal intensity across three PVC brands (0 days). Table S3: Comprehensive statistical evaluation of migration trends (one‐way ANOVA and Student's t tests) for all monitored additives in PVC films during UV aging (0, 7, and 14 days). Figure S1: Tg analysis curves for the three commercial PVC films subjected to UV aging: (A) Brand B, (B) Brand T, and (C) Brand W. The curves display the thermal degradation profiles for unexposed samples (0 days) compared with those aged for 7 and 14 days. [file RCM-40-e70048-s001.docx]

**Supplementary Material**

**Rapid detection of plasticizer migration from UV-aged PVC films by DART-HRMS**

Odilon Leite-Barbosa¹, Marcelo Ferreira Leão de Oliveira², Marcia Gomes de Oliveira², Monica Costa Padilha³, Valdir Florêncio Veiga-Junior¹*

*¹ Military Institute of Engineering (IME), Materials Engineering Department, Urca, Rio de Janeiro - RJ, 22290-270, Brazil*

*² National Institute of Technology (INT), Division of Materials, Saúde, Rio de Janeiro - RJ, 20081-312, Brazil*

*³ Brazilian Doping Control Laboratory (LBCD), Chemistry Institute, Federal University of Rio de Janeiro (UFRJ), Rio de Janeiro - RJ,* *21941-598, Brazil*

*Corresponding author: E-mail address: valdir.veiga@gmail.com (V. F. Veiga-Junior)

**Table S1**. Detailed DART-HRMS data for diethyl phthalate (DEP) (C12H14O4; theoretical *m/z* [M+H]+: 223.0965).

| Sample | Measured m/z | Mass Error (ppm) | Mean Intensity (counts) | Std. Dev. (counts) |
| --- | --- | --- | --- | --- |
| Condition: 250 °C / 50 V |  |  |  |  |
| B1 (0 days) | 223.0962 | 1.25 | 51796 | 1676 |
| B2 (7 days) | 223.0960 | 2.12 | 76816 | 1659 |
| B3 (14 days) | 223.0964 | 0.37 | 22595 | 2921 |
| T1 (0 days) | 223.0962 | 1.17 | 110602 | 54049 |
| T2 (7 days) | 223.0961 | 1.67 | 45161 | 815 |
| T3 (14 days) | 223.0965 | 0.46 | 23590 | 1510 |
| W1 (0 days) | 223.0962 | 1.19 | 44566 | 8390 |
| W2 (7 days) | 223.0961 | 1.67 | 86757 | 2679 |
| W3 (14 days) | 223.0965 | 0.13 | 17372 | 2017 |
| Condition: 350 °C / 50 V |  |  |  |  |
| B1 (0 days) | 223.0951 | 6.02 | 262898 | 76332 |
| B2 (7 days) | 223.0953 | 5.18 | 117772 | 2431 |
| B3 (14 days) | 223.0962 | 1.49 | 135953 | 33736 |
| T1 (0 days) | 223.0953 | 5.13 | 207909 | 2564 |
| T2 (7 days) | 223.0952 | 5.55 | 72828 | 6398 |
| T3 (14 days) | 223.0961 | 1.58 | 85995 | 15219 |
| W1 (0 days) | 223.0956 | 3.77 | 143242 | 32017 |
| W2 (7 days) | 223.0951 | 6.15 | 193084 | 14659 |
| W3 (14 days) | 223.0963 | 0.97 | 92816 | 2397 |
| Condition: 500 °C / 50 V |  |  |  |  |
| B1 (0 days) | 223.0955 | 4.58 | 176301 | 34502 |
| B2 (7 days) | 223.0954 | 4.76 | 94949 | 19358 |
| B3 (14 days) | 223.0963 | 0.71 | 26725 | 5849 |
| T1 (0 days) | 223.0954 | 4.95 | 64476 | 13305 |
| T2 (7 days) | 223.0955 | 4.63 | 34416 | 1318 |
| T3 (14 days) | 223.0961 | 1.86 | 20014 | 1594 |
| W1 (0 days) | 223.0953 | 5.34 | 110714 | 2368 |
| W2 (7 days) | 223.0953 | 5.24 | 44090 | 10274 |
| W3 (14 days) | 223.0958 | 3.01 | 18796 | 1559 |
| Condition: 250 °C / 350 V |  |  |  |  |
| B1 (0 days) | 223.0963 | 0.95 | 28150 | 880 |
| B2 (7 days) | 223.0964 | 0.44 | 47903 | 24819 |
| B3 (14 days) | 223.0963 | 0.74 | 19072 | 1812 |
| T1 (0 days) | 223.0963 | 0.73 | 39003 | 10610 |
| T2 (7 days) | 223.0965 | 0.48 | 12571 | 3438 |
| T3 (14 days) | 223.0964 | 0.40 | 20252 | 11356 |
| W1 (0 days) | 223.0964 | 0.55 | 25989 | 3219 |
| W2 (7 days) | 223.0964 | 0.25 | 34711 | 287 |
| W3 (14 days) | 223.0964 | 0.20 | 9225 | 6315 |
| Condition: 350 °C / 350 V |  |  |  |  |
| B1 (0 days) | 223.0965 | 0.16 | 18572 | 557 |
| B2 (7 days) | 223.0964 | 0.44 | 52511 | 35238 |
| B3 (14 days) | 223.0964 | 0.64 | 24042 | 12367 |
| T1 (0 days) | 223.0963 | 0.68 | 26877 | 3223 |
| T2 (7 days) | 223.0965 | 0.24 | 26900 | 93 |
| T3 (14 days) | 223.0964 | 0.61 | 12057 | 2118 |
| W1 (0 days) | 223.0962 | 1.13 | 25262 | 1641 |
| W2 (7 days) | 223.0965 | 0.16 | 23678 | 4786 |
| W3 (14 days) | 223.0964 | 0.49 | 6906 | 3334 |
| Condition: 500 °C / 350 V |  |  |  |  |
| B1 (0 days) | 223.0982 | 7.58 | 27654 | 2506 |
| B2 (7 days) | 223.0983 | 8.21 | 24723 | 8137 |
| B3 (14 days) | 223.0964 | 0.28 | 16407 | 1793 |
| T1 (0 days) | n.d. | n.d. | n.d. | n.d. |
| T2 (7 days) | 223.0978 | 5.84 | 45768 | 22806 |
| T3 (14 days) | 223.0964 | 0.62 | 2566 | 14 |
| W1 (0 days) | 223.0965 | 0.23 | 29397 | 9512 |
| W2 (7 days) | 223.0984 | 8.37 | 22938 | 2653 |
| W3 (14 days) | n.d. | n.d. | n.d. | n.d. |

n.d.: not detected (< LOD).

**Figure S1.** Tg analysis curves for the three commercial PVC films subjected to UV aging: (A) Brand B; (B) Brand T; and (C) Brand W. The curves display the thermal degradation profiles for unexposed samples (0 days) compared to those aged for 7 and 14 days.

**(B)**


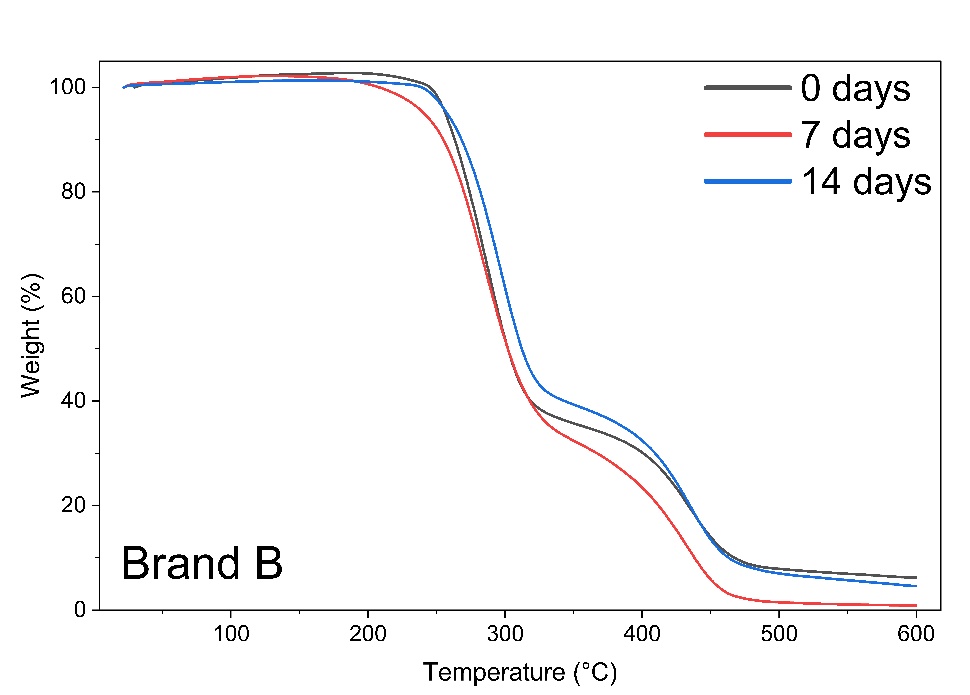

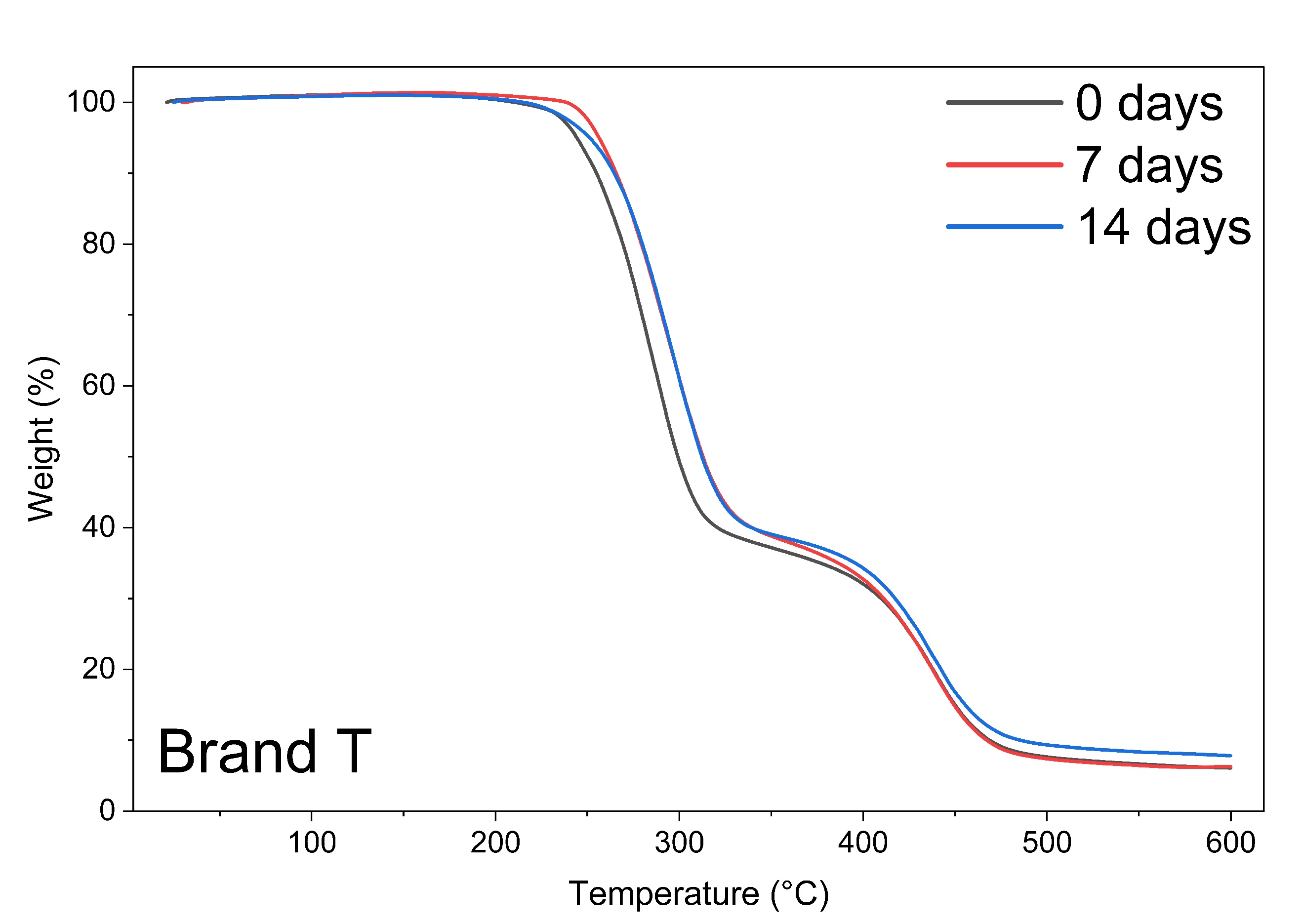

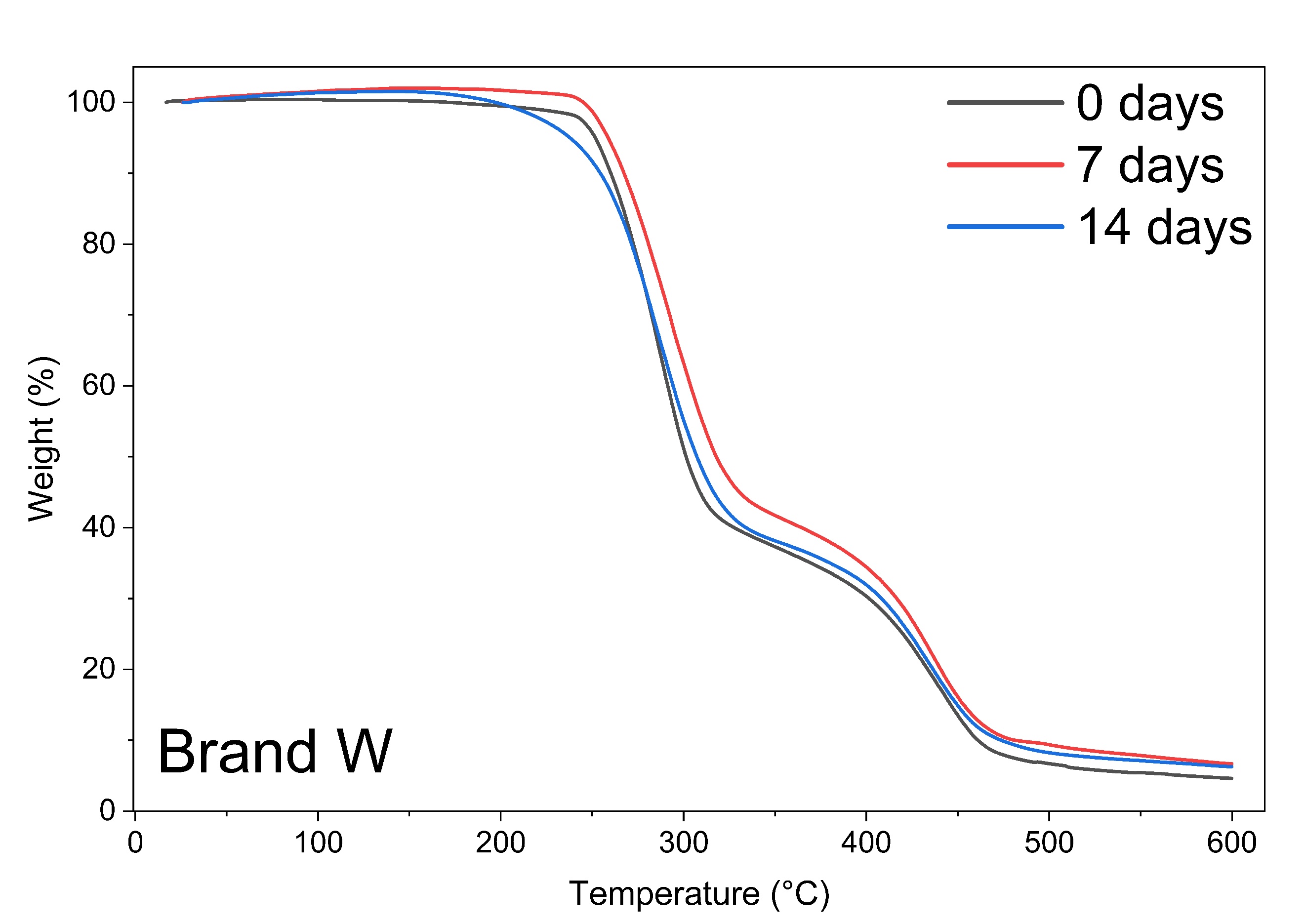


**(C)**

**(A)**

**Table S2**. Statistical optimization analysis (Two-Way ANOVA and Student’s t-tests) for Dibutyl phthalate (DBP/DIBP) signal intensity across three PVC brands (0 days).

| Statistical Test / Source of Variation | Brand B (Statistical Values) | Brand T (Statistical Values) | Brand W (Statistical Values) |
| --- | --- | --- | --- |
| Two-Way ANOVA |  |  |  |
| Grid Voltage (*df*=1) | *F* = 325.86 (**p < 0.001**) | *F* = 24.34 (**p < 0.001**) | *F* = 10.12 (**p = 0.008**) |
| Gas Temperature (*df*=2) | *F* = 52.88 (**p < 0.001**) | *F* = 5.60 (**p = 0.019**) | *F* = 7.36 (**p = 0.008**) |
| Interaction (Volt x Temp) (*df*=2) | *F* = 194.67 (**p < 0.001**) | *F* = 1.05 (*p* = 0.380) | *F* = 4.91 (**p = 0.028**) |
| Student's t-test (at 50 V) |  |  |  |
| 250 °C vs. 350 °C | **p < 0.001** | *p* = 0.301 | **p = 0.002** |
| 350 °C vs. 500 °C | **p = 0.010** | **p = 0.030** | *p* = 0.110 |
| 250 °C vs. 500 °C | **p < 0.001** | *p* = 0.610 | *p* = 0.420 |
| Student's t-test (at 350 V) |  |  |  |
| 250 °C vs. 350 °C | **p = 0.028** | *p* = 0.899 | *p* = 0.385 |
| 350 °C vs. 500 °C | p = 0.825 | p = 0.004 | p = 0.380 |
| 250 °C vs. 500 °C | p = 0.002 | p = 0.002 | p = 0.900 |

Note: df = degrees of freedom; F = F-statistic; p = p-value. Analyses were performed with a 95% confidence level (α = 0.05). Values in bold indicate statistical significance (p ≤ 0.05).

**Table S3**. Comprehensive statistical evaluation of migration trends (One-Way ANOVA and Student’s t-tests) for all monitored additives in PVC films during UV aging (0, 7, and 14 days).

| Analyte / Statistical Test | Brand B (Statistical Values) | Brand T (Statistical Values) | Brand W (Statistical Values) |
| --- | --- | --- | --- |
| 1. Dibutyl phthalate (DBP/DIBP) |  |  |  |
| One-Way ANOVA (*F*, *p*) | *F* = 7.91 (**p = 0.021**) | *F* = 16.67 (**p = 0.004**) | *F* = 63.50 (**p < 0.001**) |
| t-test: 0 days vs. 7 days | *p* = 0.160 | *p* = 0.070 | **p = 0.005** |
| t-test: 7 days vs. 14 days | *p* = 0.110 | **p = 0.005** | *p* = 0.700 |
| t-test: 0 days vs. 14 days | *p* = 0.070 | *p* = 0.050 | **p = 0.002** |
|  |  |  |  |
| 2. Diethyl phthalate (DEP) |  |  |  |
| One-Way ANOVA (*F*, *p*) | *F* = 8.44 (**p = 0.018**) | *F* = 16.92 (**p = 0.003**) | *F* = 12.75 (**p = 0.007**) |
| t-test: 0 days vs. 7 days | *p* = 0.060 | **p = 0.020** | *p* = 0.100 |
| t-test: 7 days vs. 14 days | *p* = 0.260 | *p* = 0.100 | **p = 0.020** |
| t-test: 0 days vs. 14 days | *p* = 0.090 | **p = 0.040** | *p* = 0.070 |
|  |  |  |  |
| 3. DEHP Group |  |  |  |
| One-Way ANOVA (*F*, *p*) | F = 2.64 (p = 0.151) | F = 4.18 (p = 0.073) | F = 4.55 (p = 0.063) |
| t-test: 0 days vs. 7 days | p = 0.090 | p = 0.110 | p = 0.470 |
| t-test: 7 days vs. 14 days | p = 0.320 | p = 0.120 | p = 0.140 |
| t-test: 0 days vs. 14 days | p = 0.200 | p = 0.910 | p = 0.090 |
|  |  |  |  |
| 4. Diisononyl phthalate (DINP) |  |  |  |
| One-Way ANOVA (*F*, *p*) | F = 1.39 (p = 0.320) | *F* = 14.65 (**p = 0.005**) | *F* = 12.19 (**p = 0.008**) |
| t-test: 0 days vs. 7 days | p = 0.720 | *p* = 0.230 | *p* = 0.320 |
| t-test: 7 days vs. 14 days | p = 0.180 | **p = 0.020** | *p* = 0.050 |
| t-test: 0 days vs. 14 days | p = 0.370 | **p = 0.030** | *p* = 0.050 |
|  |  |  |  |
| 5. Diisodecyl phthalate (DIDP) |  |  |  |
| One-Way ANOVA (*F*, *p*) | F = 1.77 (p = 0.249) | *F* = 9.20 (**p = 0.015**) | *F* = 17.14 (**p = 0.003**) |
| t-test: 0 days vs. 7 days | p = 0.790 | *p* = 0.340 | *p* = 0.290 |
| t-test: 7 days vs. 14 days | p = 0.220 | *p* = 0.070 | **p = 0.040** |
| t-test: 0 days vs. 14 days | p = 0.170 | *p* = 0.080 | **p = 0.040** |
|  |  |  |  |
| 6. Dimethyl sulfoxide (DMSO) |  |  |  |
| One-Way ANOVA (F, p) | *F* = 18.23 (**p = 0.003**) | *F* = 28.34 (**p < 0.001**) | *F* = 9.36 (**p = 0.014**) |
| t-test: 0 days vs. 7 days | *p* = 0.080 | *p* = 0.500 | *p* = 0.930 |
| t-test: 7 days vs. 14 days | **p = 0.020** | **p = 0.001** | **p = 0.050** |
| t-test: 0 days vs. 14 days | **p = 0.050** | **p = 0.020** | **p = 0.020** |

Note: Analyses were performed with a 95% confidence level (α = 0.05). One-way ANOVA was calculated with degrees of freedom df_between = 2 and df_within = 6 (n = 3 replicates). Values in bold (p ≤ 0.05) indicate statistically significant differences between aging times.
